# Response to Comment on “Symptoms, Imaging Features, Treatment Decisions, and Outcomes of Patients with Top of the Basilar Artery Syndrome: Experiences from a Comprehensive Stroke Center”

**DOI:** 10.1007/s12028-025-02265-6

**Published:** 2025-04-23

**Authors:** Franziska Lieschke

**Affiliations:** Department of Neurology, University Hospital, Goethe University Frankfurt, Schleusenweg 2-16, 60528 Frankfurt Am Main, Germany

Dear Editor,

We appreciate Dr. Memon’s thoughtful critique of our study, “Symptoms, Imaging Features, Treatment Decisions, and Outcomes of Patients with Top of the Basilar Artery Syndrome: Experiences from a Comprehensive Stroke Center.” We welcome the opportunity to clarify key points and address concerns raised.

As stated in the introduction of our article [1], although magnetic resonance imaging (MRI) is superior for detecting posterior circulation strokes, its use in emergency settings is often limited. Patients may struggle to remain still, MRI-compatible monitors are required, and many protocols necessitate extra staff, such as anesthesiologists. These factors can delay diagnosis. Thus, computed tomography/computed tomography angiography (CT/CTA) remains the common choice in real-world practice, providing rapid, actionable insights. Accordingly, the majority of our patients received CT/CTA (62.5%), whereas the MRI group encompassed 37.5%. CTA provides fast information about vessel occlusion and collateral status. Furthermore, it may also identify rare causes of top of the basilar artery syndrome (TOBS), such as unruptured giant basilar artery aneurysm, i.e., Castaigne syndrome [2].

In the stroke unit, standard laboratory tests include a complete blood count, fasting lipid profiles, coagulation panels, and glycated hemoglobin levels, all of which help to identify stroke risk factors and etiology. Table 2 of our article outlines the presumed etiology based on the Trial of Org 10,172 in Acute Stroke Treatment (TOAST) classification, which was determined after a thorough workup [1]. We recognize the potential value of incorporating more comprehensive laboratory tests, such as the biomarkers alarmins and cfDNA, alongside imaging and clinical data. As cfDNA can be actively released from hypoxic cells, identifying its source could help to differentiate between cardioembolic thrombi and atherosclerosis [3–6]. This approach could help to identify the underlying etiology, providing deeper insights into its mechanisms and enabling more personalized treatment strategies.

The challenge of delayed diagnosis in posterior strokes is well documented, often due to atypical presentations and lower awareness [7, 8]. As discussed in our article, the majority of our patients (53%) were transfer patients. The patients’ records provided from the primary stroke centers often lacked important information, or times were inaccurately remembered. Although our study did not systematically capture onset-to-treatment times, it accurately reflects real-world treatment decisions. As a retrospective analysis, it does not allow for proof of treatment efficacies but rather identifies associations, adds descriptive information and serves as a basis for hypothesis generation.

Collateral circulation, a key prognostic factor [9–11], was not the primary focus of our analysis, but we recognize its importance and support further research specifically addressing TOBS, as most studies on this topic examined basilar artery occlusions in general (often referring to the occlusion of the middle part of the vessel), and fewer examined the occlusion of the distal part of the basilar artery [12].

The concern regarding symptomatic intracranial hemorrhage (sICH) is justified but should be put into context. We reviewed the data set, and sICH per the SITS-MOST definition (a type 2 parenchymal hemorrhage with deterioration in the National Institutes of Health Stroke Scale score of ≥ 4 points or death) occurred in 2.1% (1.2% in IVT ± MT-treated patients), reinforcing the safety of these interventions even in late time windows. Minor hemorrhagic transformations (hemorrhagic infarctions type 1 or 2) were observed in six patients (6.3%) but did not worsen outcomes. Minor contrast extravasation/subarachnoid hemorrhage during MT was observed in four patients (4.2%), which remained stable on follow-up imaging. The affected patients improved significantly despite the findings.

In conclusion, based on insights into the clinical course of TOBS, we highly welcome further investigations incorporating laboratory markers, collateral assessment, precise timing data, and long-term outcomes to advance the field. We hope this response clarifies our approach and provides a foundation for future research in this time-critical area of neurovascular care.

Sincerely,

Dr. Franziska Lieschke.
